# Case Report: Right aortic arch with isolation of left brachiocephalic artery and ventricular septal defect

**DOI:** 10.3389/fcvm.2024.1381222

**Published:** 2024-04-22

**Authors:** Gang Wang, Jieqiong Wang, Hongjie Zhang, Hui Wang, Qiang Meng, Linhong Song, Gengxu Zhou

**Affiliations:** ^1^ The Second School of Clinical Medicine, Southern Medical University, Guangzhou, China; ^2^ Department of Pediatric Cardiac Surgery, The Seventh Medical Center of the PLA General Hospital, Beijing, China; ^3^ Department of Radiology, The Seventh Medical Center of the PLA General Hospital, Beijing, China

**Keywords:** isolation of left brachiocephalic artery, right aortic arch, aortic arch anomaly, congenital heart defect, subclavian steal syndrome

## Abstract

Right aortic arch with isolation of left brachiocephalic artery is a rare congenital aortic arch anomaly. Herein, we reported a case of this rare anomaly with ventricular septal defect in a 9-month-old infant. We successfully reconstructed the islolated left brachiocephalic artery and repaired the ventricular septal defect in one stage.

## Introduction

Right aortic arch (RAA) with isolation of the left brachiocephalic artery (ILBA) is a very rare congenital anomaly. This anomaly is defined as the left brachiocephalic artery (LBA) loses its attachment to the aorta and is connected with the pulmonary artery(PA) via a ductus arteriosus(DA) (1–3). Reconstructing continuity between the LBA and the aortic arch can be difficult when the two vessels are far apart. In this report, we used a pedicle flap of ascending aorta as the posterior wall and a bovine pericardial patch as the anterior wall to establish the continuity between the LBA and the arotic arch.

## Case report

**Figure 1:**
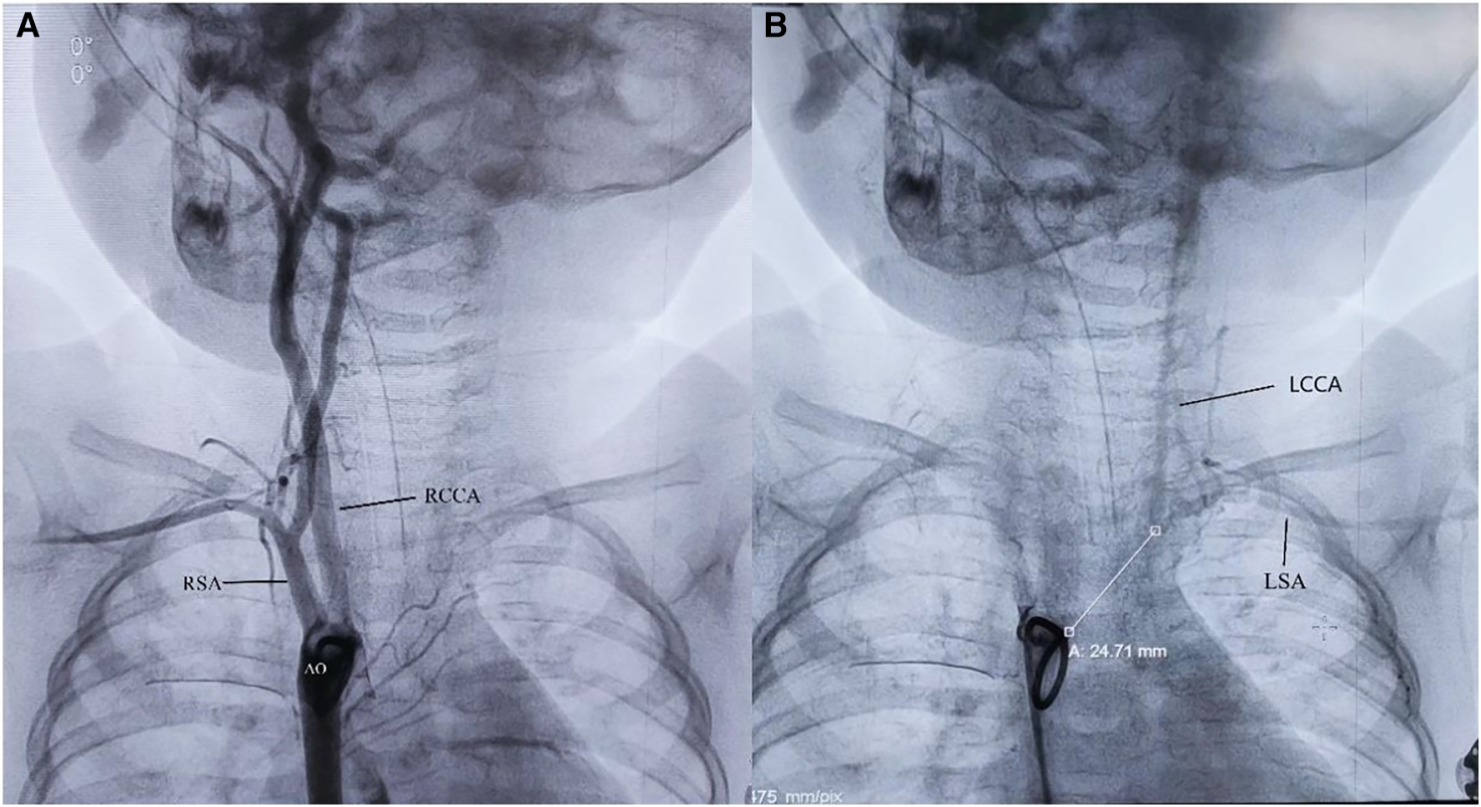
(**A**) The aortic angiogram demonstrated a right aortic arch with only two arch branches. (**B**) The LBA have no connection with the aortic arch and was delayed filled by retrograde flow from the cerebral circulation. AO, aorta; RCCA, right common carotid artery; RSA, right subclavian artery; LCCA, left common carotid artery; LSA, left subclavian artery; white line: The distance between the LBA and AO is 24.71 millimeters.

A 9-month-old male infant was referred to our hospital due to recurrent respiratory tract infection. The patient was 7.5 kilogram and had normal growth and development. Clinical examination findings included a precordial grade 3/6 systolic murmur, left radial artery and left carotid artery pulses were not palpable. Both upper limbs were the same size. Echocardiography showed a 6 mm perimembranous ventricular septal defect (VSD) and the right aortic arch, with no LBA arising from the aortic arch. The computed tomographic angiography showed the right aortic arch and its only two branches, the right common carotid artery and the right subclavian artery. To identify the branch of the aortic arch, we performed cardiac catheterization for the patient. The aortic angiogram demonstrated a right aortic arch with only two arch branches. The first branch was the right common carotid artery followed by the right subclavian artery. The LBA have no connection with the aortic arch and was delayed filled by retrograde flow from the cerebral circulation (Figure 1). Considering the long-term development of the cerebral and the left upper extremity, we decided to perform VSD repair and reconstruct the LBA in one stage.

**Figure 2:**
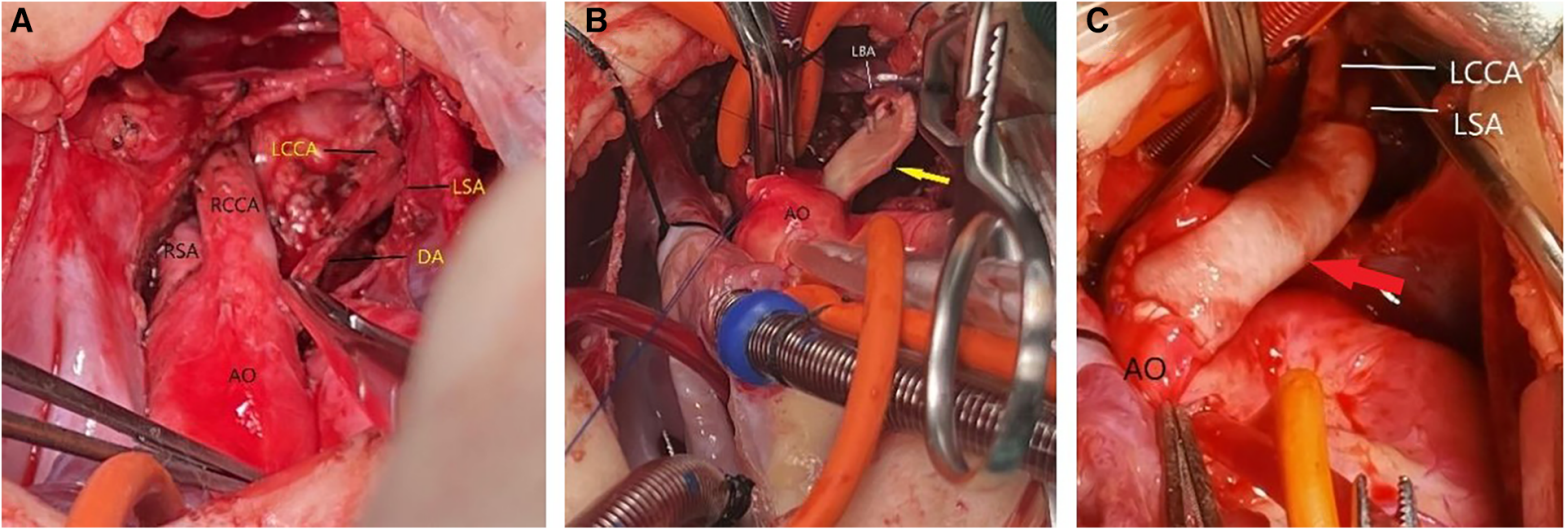
(**A**) The LBA was connected to the main pulmonary artery via the left ligamentum arteriosum. (**B**) A pedicle flap was created in the anteriolateral wall of the ascending aorta. The aortic flap was turned upward and anastomosed with the posterior wall of the LBA. (**C**) A bovine pericardial patch was used as the anterior wall to connect the aortic arch with the LBA. AO, aorta; RCCA, right common carotid artery; RSA, right subclavian artery; LCCA, left common carotid artery; LSA, left subclavian artery; DA, ductus arteriosus; LBA, left brachiocephalic artery. yellow arrow: a pedicle flap of the ascending aorta. red arrow: a bovine pericardial patch.

**Figure 3:**
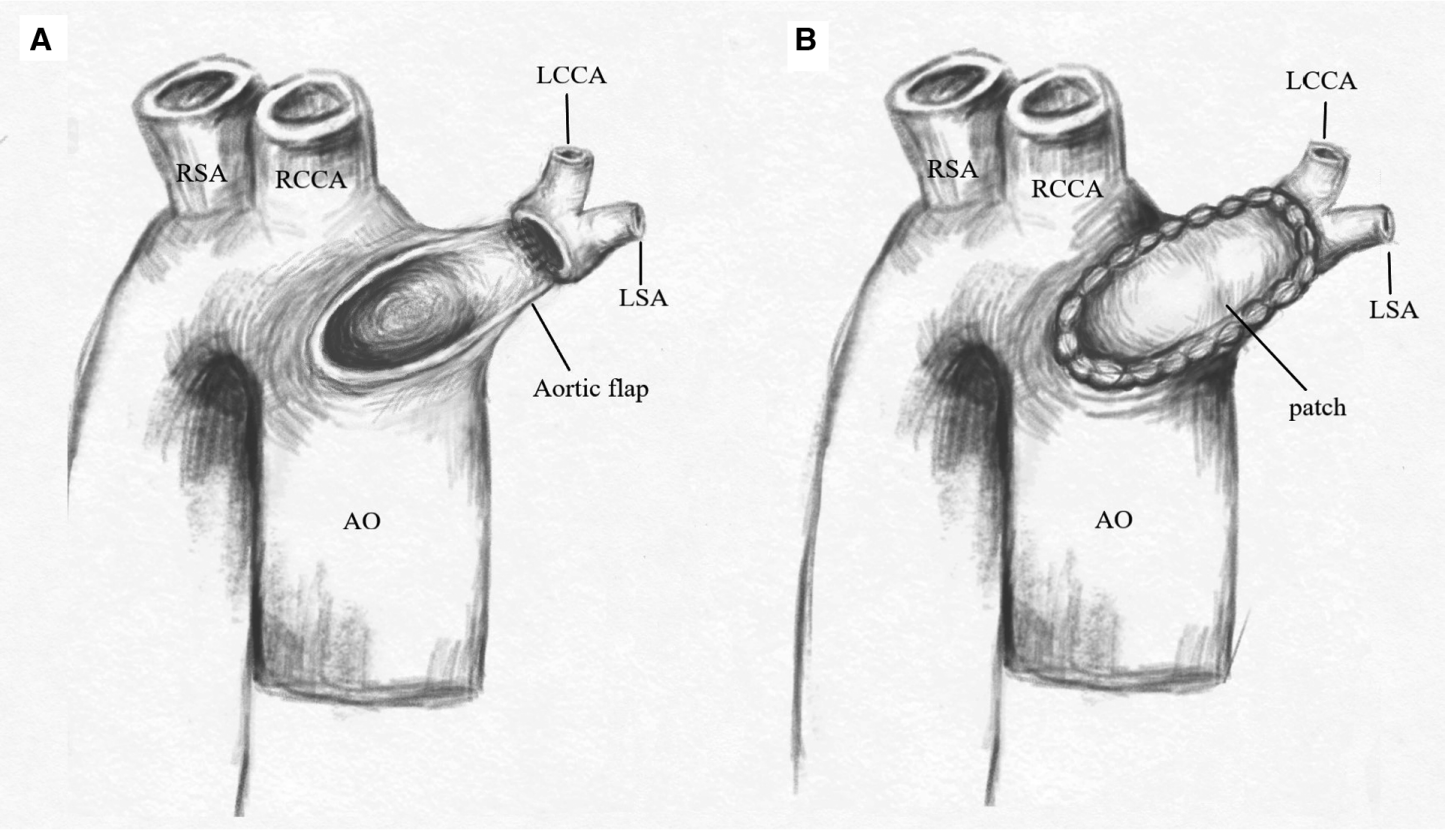
(**A**) A pedicle flap was created in the anteriolateral wall of the ascending aorta. The aortic flap was turned upward and anastomosed with the posterior wall of the LBA. (**B**) A bovine pericardial patch was used as the anterior wall to connect the aortic arch with the LBA. AO, aorta; RCCA, right common carotid artery; RSA, right subclavian artery; LCCA, left common carotid artery; LSA, left subclavian artery.

**Figure 4:**
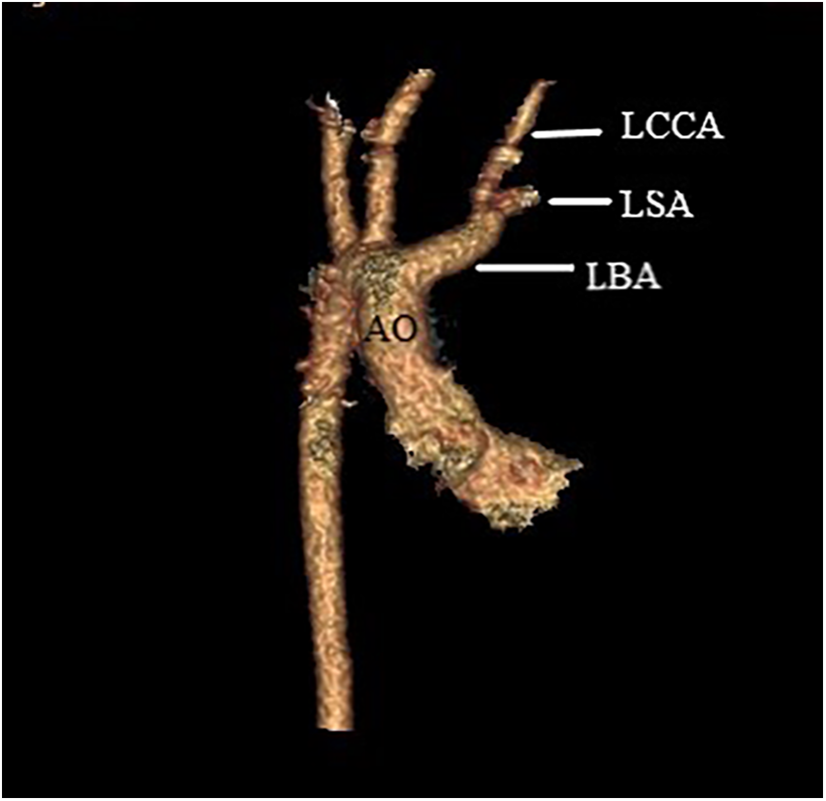
Postoperative computed tomographic angiography showed that the reconstructed LBA was patent. AO, aorta; LCCA, left common carotid artery; LSA, left subclavian artery; LBA, left brachiocephalic artery.

A median sternotomy was performed. During the operation, we found that the LBA was connected to the main pulmonary artery via the left ligamentum arteriosum (Figure 2). The LBA and its branches, as well as the ascending aorta were extensively mobilized. Cardiopulmonary bypass was established by bicaval cannulation and aortic cannulation. The aortic cannula was placed near the right common carotid artery origin. The VSD was repaired with a bovine pericardial patch via a right atriotomy. The left ligamentum arteriosum was divided. After the residual ductal tissues on the side of the LBA were removed, there was a significant distance between the isolated LBA and the aortic arch. A pedicle flap was created in the anteriolateral wall of the ascending aorta. The aortic flap was turned upward and anastomosed with the posterior wall of the LBA, and a bovine pericardial patch was used as the anterior wall to connect the aortic arch with the LBA (Figures 2, 3). Postoperatively, the patient had good left radial artery pulse, and computed tomographic angiography showed that the reconstructed LBA was patent (Figure 4). During the 6 months follow-up, the patient was asymptomatic and had normal development.

## Discussion

Right aortic arch (RAA) with ILBA is a rare anatomic variation of aortic arch anomaly, in which the LBA loses its attachment to the aorta and abnormally connect with the PA via a ductus arteriosus. To date, most reports of this anomaly were case reports, and most cases were associated with intracardiac malformations (3–9) (Table 1). The clinical presentation, management, and prognosis of the patients with ILBA depend on anatomical characteristics and whether it is associated with other malformations. Rad (8) and colleagues proposed a novel anatomic-clinical-prognostic classification of ILBA, classifying it into three types: single-steal type with single source of steal from LBA through the left subclavian artery and with no connection to PA, double-steal type with double sources of steal from LBA through left subclavian artery and patent ductus arteriosus (PDA), triple-steal type with triple sources of steal from LBA through left subclavian artery and bilateral PDAs. According to the above classification, our case belonged to the single-steal type. In our case, the absence of pulse in the left radial artery and left carotid artery, with the presence of a right aortic arch with two arch branches led us to suspect this anomaly, and aorta angiography confirmed the diagnosis.

**Table 1:** Cases characteristics in references.

Authors	Year	Age	Sex	Symptom	Physical examination	Type of ILBA	Associated anomaly	Treatment	Follow-up
Gamillscheg. et al. (6)	1999	6 months	M	NS	A grade 3/6 systolic murmur at the left sternal border with a loud single second sound	Double-steal	Trisomy 21, VSD,ASD, left PDA	Patch closure of the VSD and direct closure of the ASD, the LBA was anastomosed with the ascending aorta by interposition of an 8 mm polytetrafluoroethylene tube	Asymptomatic at 6 months follow-up
Gil-Jaurena. et al. (5)	2011	6 months	NS	Congestive heart failure	A systolic murmur	Single-steal	ASD	ASD was closed, LBA was reimplanted end-to-side to the ascending aorta	NS
Mangukia. et al. (9)	2014	10 years	M	Shortness of breath while playing	Pulse was weaker on the left radial artery and with a delay compared to right radial pulse	Single-steal	VSD,DCRV	VSD closure and Infundibular band resection. The LBA was not reconstructed	NS
Gowda. et al. (10)	2014	5 years	M	Weakness of left upper limb on physical exertion	Left upper limb and left carotid pulsations were absent	Single-steal	NO	LBA was reimplanted into the aorta	NS
Dubey. et al. (3)	2017	9 years	M	Dyspnea on exertion, palpitations on exertion, and failure to gain weight	Left upper limb pulses were not palpable. a grade 3 continuous murmur at the left 2nd and 3rd intercostal spaces and a soft mid-diastolic murmur at apex	Double-steal	Large PDA	Reimplantation of LBA to aortic arch and PDA ligation	NS
Schwartz. et al. (7)	2018	4 weeks	M	NS	NS	Triple-steal	CHARGE syndrome, Bilateral PDA, ASD	ASD closure, bilateral PDA ligation. The pedicle autologous aortic flap was anastomosed to the base of the LBA, creating the back wall, while a homograft patch anteriorly allowed reconstitution of the LBA to the aortic arch	At one-year follow-up, the aortic arch and the LBA were widely patent
Rad. et al. (8)	2019	3.5 years	F	NS	A grade 4/6 holosystolic murmur was heard at the left sternal border. The left carotid, left brachial, and left radial pulses were weaker than the right ones	Single-steal	VSD	VSD closure, LBA was not reimplanted	NS
Tran. et al. (4)	2020	3 weeks	M	Cyanosis while breast feeding	NS	Triple-steal	Choanal atresia, bilateral PDA	PDA ligation. The LBA was reimplanted into the ascending aorta	Normal physical development

M, male; NS, not stated; VSD, ventricular septal defect; ASD, atrial septal defect; PDA, patent ductus arteriosus; LBA, left brachiocephalic artery; DCRV, double chamber right ventricle; F, female.

The ILBA could induce subclavian artery steal phenomenon, which decrease perfusion of the cerebral and left upper extremity and affects the development of these organs. If combined with PDA, it can also lead to pulmonary artery steal and cause congestive heart failure. Therefore, we suggested that the continuity between the LBA and the aorta should be reconstructed for this anomaly, which is very important to preserve normal perfusion of the cerebral and left upper extremity and maintain organs development. Various surgical techniques for reconstructed the continuity between the LBA and the aorta had been reported in previous literature, such as direct anastomosis of IBA and aorta (4, 5, 10), using a synthetic tube to connect the LBA and the aorta (6). In our patient, there was a significant distance between the isolated LBA and the aortic arch, two vessles were unable to be anastomosed directly. However, we also did not consider using synthetic tube because it has no growth potential and could stenosis in the long term. Schwartz (7) and colleagues described a case of this anomaly using an autologous pedicle flap of ascending aorta as well as a homograft patch as the roof to recreate continuity between the aorta and left innominate artery. We drawed on this technique to reconstructed the continuity between the LBA and the aorta, and achieved satisfactory outcome. The long-term patency of the LBA needed further follow-up.

## Conclusion

Right aortic arch with isolation of left brachiocephalic artery is a rare congenital aortic arch anomaly. Absent or weak pulse of the left radial artery combined with right aortic arch and two arch branches is the clue to the diagnosis of the ILBA. Reconstructing the continuity between the LBA and the aorta is beneficial to the development of children. The technique we described in this report provides a surgical strategy for patients with two vessels far apart.

## Data Availability

The original contributions presented in the study are included in the article/**Supplementary Material**, further inquiries can be directed to the corresponding author.
